# A Multiscale Lightweight and Efficient Model Based on YOLOv7: Applied to Citrus Orchard

**DOI:** 10.3390/plants11233260

**Published:** 2022-11-27

**Authors:** Junyang Chen, Hui Liu, Yating Zhang, Daike Zhang, Hongkun Ouyang, Xiaoyan Chen

**Affiliations:** 1 College of Information Engineering, Sichuan Agricultural University, Ya’an 625000, China; 2 College of Mechanical and Electrical Engineering, Sichuan Agricultural University, Ya’an 625000, China; 3 Sichuan Key Laboratory of Agricultural Information Engineering, Ya’an 625000, China

**Keywords:** citrus, YOLOv7, attention mechanism, multi-scale fusion, lightweight

## Abstract

With the gradual increase in the annual production of citrus, the efficiency of human labor has become the bottleneck limiting production. To achieve an unmanned citrus picking technology, the detection accuracy, prediction speed, and lightweight deployment of the model are important issues. Traditional object detection methods often fail to achieve balanced effects in all aspects. Therefore, an improved YOLOv7 network model is proposed, which introduces a small object detection layer, lightweight convolution, and a CBAM (Convolutional Block Attention Module) attention mechanism to achieve multi-scale feature extraction and fusion and reduce the number of parameters of the model. The performance of the model was tested on the test set of citrus fruit. The average accuracy (mAP_@0.5_) reached 97.29%, the average prediction time was 69.38 ms, and the number of parameters and computation costs were reduced by 11.21 M and 28.71 G compared with the original YOLOv7. At the same time, the Citrus-YOLOv7 model’s results show that it performs better compared with the current state-of-the-art network models. Therefore, the proposed Citrus-YOLOv7 model can contribute to solving the problem of citrus detection.

## 1. Introduction

Citrus is a Rutaceae, a citrus plant mainly distributed between 16°–37° north latitude, and is a common tropical and subtropical evergreen fruit tree [1]. Citrus fruit is loved by people all over the world because of its sweet and sour pulp and rich nutrition [2], and is regarded as a precious fruit. However, in the current citrus production process, there is a strict time limit for fruit picking and the demand for labor is large, therefore the impact of the labor shortage is more serious. It is gratifying and worrying that the annual production of citrus, the world’s first fruit, is expected to exceed 160 million tons in 2022 [3,4], which means that labor costs will also increase significantly. With the rapid development of artificial intelligence, the work of fruit picking may be replaced by agricultural robots [5].

In recent years, the field of artificial intelligence in agriculture has remained in its infancy. To realize the commercialization of agricultural robots, the following three major problems must be solved. The first is that the detection accuracy needs to be improved, the second is the inference speed of the model, and the last is the lightweight deployment of the model [6]. As an important part of the development of agricultural robots, the detection and recognition of citrus fruits is of great significance. Over the past decades of rapid development of deep learning, many excellent modules and networks have been proposed, but most of them remain in the theoretical stage, lack practical applications, or fail to fully solve the above problems, and need further improvement. In terms of fruit detection, many researchers have achieved good research results, and the research in this paper must be based on previous research.

As a part of model training, preprocessing work is of great significance in improving model capability and alleviating overfitting. Kukreja V et al. proposed a dense CNN network, which provided an idea for citrus quality detection by designing data augmentation and preprocessing techniques [7]. Sa I et al. [8] explored a multi-modal object detection method by fusing captured RGB images with near-infrared spectral images, which improved the detection effect of fruits. Bini Darwin et al. [9] believed that the application of normalized histograms could unify the latitude of image samples and ultimately speed up model training. In the actual orchard environment, the traditional machine vision detection accuracy is often unsatisfactory due to the different degrees of occlusion between leaves, fruits, and branches [10]. Data-driven deep learning technology can solve the shortcomings of traditional technology. Liu G et al. [11] integrated a dense architecture based on YOLOv3 to achieve the effect of functional reuse, and were the first to replace the rectangular frame with a circular detection frame, which left a deep impression. Qi J et al. [12] learned from the human visual attention mechanism and added the SE module [13] to the network, which effectively proposed key features and achieved a detection accuracy of 94.10%, which provides a certain reference value for the work of this paper. Liu et al. [14] used MobileNetv2 as the backbone network for citrus disease detection, which reduced the scale of the model and contributed to solving the model’s lightweight problem. QIU W et al. [15] studied a model compression method based on knowledge distillation, which achieved relatively optimized results in terms of reducing parameters and improving detection speed. The work of the above scholars has promoted the development of fruit detection to a certain extent, and they have shown their thinking in terms of accuracy, reasoning speed, and lightweight. However, the real orchard situation is complex and requires a comprehensive solution to all three problems. Therefore, there is still room for improvement in current fruit detection work.

To achieve the unmanned picking of citrus, the problem is the accurate and rapid identification and positioning of citrus fruits in the natural environment [16], where it is difficult to avoid the problems listed above. In this paper, we make and present a dataset of citrus fruits collected in citrus orchards, using the state-of-the-art object detector—YOLOv7 [17], based on the CBAM attention mechanism [18], GhostConv convolution module [19], while adding a small object detection box to the model. Experiments on the dataset proposed in this paper demonstrate that these improvements are beneficial to improving the detection accuracy and inference speed, and achieving a lightweight model.

## 2. Related Work

### 2.1. YOLOv7: Excellent Aggregator

**Figure 1:**
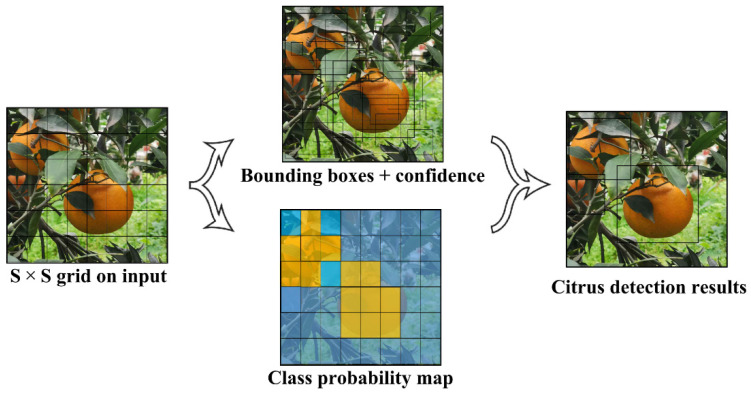
YOLO network detection process.

YOLOv7 [17] is the latest work of the YOLO (You Only Look Once) series, and it is also the most advanced object detection model. In 2015, YOLOv1 [20] was proposed, and the single-stage detection algorithm first appeared in people’s fields of vision. It effectively solved the shortcoming of the slow reasoning speed of the two-stage detection network and maintained a good effect on detection accuracy. The specific operation is shown in Figure 1. YOLOv3 [21] was an improved version of the previous work. Its biggest feature was the introduction of the residual module Darknet-53 and FPN architecture, which predicted objects at three different scales and realized multi-scale fusion. Since then, YOLOv4 [22] and YOLOv5 [23] have added many tricks based on version 3. In 2022, YOLOv7 was born, and it innovatively proposed the Extended-ELAN architecture, which can improve the self-learning ability of the network without destroying the original gradient path. In addition, it also adopts a cascade-based model scaling method, so that models of corresponding scales can be generated for practical tasks to meet detection requirements.

### 2.2. Attention Mechanism: Selectively Paying Attention to Information

The attention mechanism first originated from the study of human vision. It simulates the phenomenon that humans selectively focus on some visible information and ignore other information to reasonably utilize limited visual processing resources. An attention mechanism is introduced through the field of deep learning, mainly by selecting only a part of the input information or assigning different weights to different parts of the input information, to solve the problem of information redundancy.

In the process of exploring the application of the attention mechanism in computer vision, many excellent works have emerged. CBAM (Convolutional Block Attention Module) is a plug-and-play attention module proposed by Woo S et al. [18] in 2018, which innovatively integrates channel attention with spatial attention, allowing the network to focus on important features and suppress unnecessary features. The biggest difference between CBAM and other modules is that it focuses on both channel and spatial dimensions and achieves the best efficiency.

Suppose the input feature map of the network is: $F\in R^{C\;\times \;H\;\times \;W}$, where F refers to the input feature graph, and R represents the set of real numbers, which represents the feature graph with the number of channels C, the height H, and the width W. Then the channel feature map is generated after passing through the first channel attention module: $M_{c}\in R^{C\;\times 1\times 1}$. The spatial feature map is generated after passing through the second spatial attention module: $M_{s}\in R^{1\times \;H\;\times \;W}$. The formula can be expressed as follows:

(1) $$\;F^{\prime }=M_{c}\left(F\right)⊗F$$

(2) $$F^{\prime \prime }=M_{s}\left(\;F^{\prime }\right)⊗\;F^{\prime }$$

The channel attention module uses the spatial dimension method of compressing the input feature map and simultaneously applies the AvgPool and MaxPool methods, which can effectively calculate the weight attention assigned to the channel dimension. The formula is as follows:

(3) $$M_{c}\left(F\right)=\sigma \left(\mathrm{MLP}\left(\mathrm{AvgPool}\left(F\right)\right)+\mathrm{MLP}\left(\mathrm{MaxPool}\left(F\right)\right)\right)$$

$$=\sigma \left(W_{1}\left(W_{0}\left(F_{\mathrm{avg}}^{c}\right)\right)+W_{1}\left(W_{0}\left(F_{\max}^{c}\right)\right)\right)$$

Among them, σ represents the Sigmoid function, $W_{0}\in R^{\frac{C}{r}\times \;C}$, $W_{1}\in R^{C\;\times \frac{C}{r}}$, where $W_{0}$ are activated by the ReLu (Rectified Linear Unit) function. MLP is a multi-layer perceptron with a hidden layer, and its operation weight is determined by${\;W}_{0}$ and $W_{1}$.

The spatial attention module focuses on the location of the information in the image, which is supplemented by the previous module. Computationally, it first adopts AvgPool and MaxPool operations on the channel axis and concatenates them into a meaningful feature descriptor. The two pooling operations will aggregate the channel information of a feature map to generate 2D maps. Finally, the convolution operation is performed by the convolution layer to obtain the corresponding spatial feature map. The specific formula is as follows:

(4) $$M_{s}\left(F\right)=\sigma \left(f^{7x7}\left(\left[\mathrm{AvgPool}\left(F\right);\;\mathrm{MaxPool}\left(F\right)\right]\right)\right)$$

$$=\sigma \left(f^{7x7}\left(\left[F_{\mathrm{avg}}^{s};{\;F}_{\max}^{s}\right]\right)\right)$$

In the formula, σ represents the Sigmoid function, $f^{7\times 7}$ represents the 7 × 7 convolution kernel, and $F_{\mathrm{avg}}^{s},F_{\max}^{s}\in R^{1\times \;H\;\times \;W}$.

**Figure 2:**
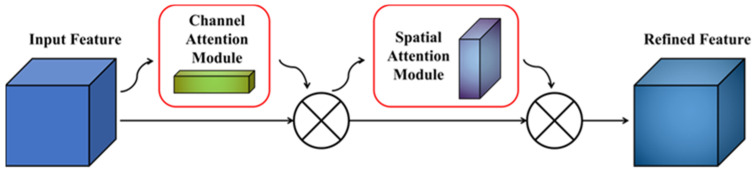
CBAM algorithm implementation flowchart.

Based on the excellent performance of CBAM, this paper will insert the CBAM attention module into the backbone network structure of Yolov7 to improve the detection accuracy of the network, as shown in Figure 2.

### 2.3. GhostConv: Lighter Convolution Module

The traditional feature extraction operation has the problem of possessing a large number of parameters. The reason for this is that multiple convolution kernels are stacked to perform convolution mapping for all the channels of the input feature map, which consumes a lot of computational resources. Although this operation can produce rich feature maps, it also inevitably causes waste. In response to this situation, some scholars have proposed some network optimization schemes, such as ShuffleNet [24] and MobileNet [25], which alleviate the bloated model to a certain extent, but the required 1 × 1 convolution will still take up part of the computational resources.

**Figure 3:**
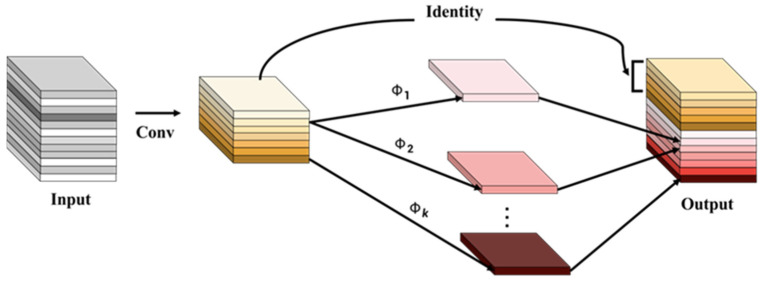
Ghost convolution schematic diagram.

The GhostConv convolution module is a good alternative to traditional convolution, as shown in Figure 3. It first extracts feature information using a small number of convolution kernels, then uses a cheap linear transformation to reduce the learning cost of non-key features, and finally generates corresponding feature maps through a concatenation operation. In general, GhostConv transforms the traditional convolution operation into a two-step process. In the first step, a small number of original feature maps are generated, and the formula is as follows:

(5) $$y_{\mathrm{ij}}=\Phi_{i,j}\left(y_{i}^{\prime }\right),\;\forall i=1,\cdots ,m,\;j=1,\cdots ,s,$$

Among them $\Phi_{i,j}$ is the Ghost operation, where $y_{\mathrm{ij}}\;$represents the channel feature map generated after the operation.

In the second step, linear cheap transformation is used to generate the final feature map. Experiments show that it has a better ability than a lightweight neural network, and can be used to solve the problem of lightweight modelling.

### 2.4. Small Object Detection

In the real world, small object detection can be seen everywhere, which is also a hot topic in the field of computer vision. In this paper, based on the definition of the MS COCO dataset [26], a small object is defined as an object with a resolution of fewer than 32 pixels × 32 pixels. The development of small object detection is difficult mainly because of some technical difficulties, such as fewer effective features, uneven samples, and the high accuracy required for the positioning and aggregation phenomenon [27]. In previous studies, multi-scale learning and context learning [28] were generally adopted to improve the detection ability of small objects. In addition, the strategy of optimizing the loss function [29] also improved the detection effect. In this experiment, there are some small objects in the citrus dataset, so it is necessary to optimize the algorithm for small objects to improve the detection ability.

## 3. Materials and Methods

### 3.1. Data Acquisition

**Figure 4:**
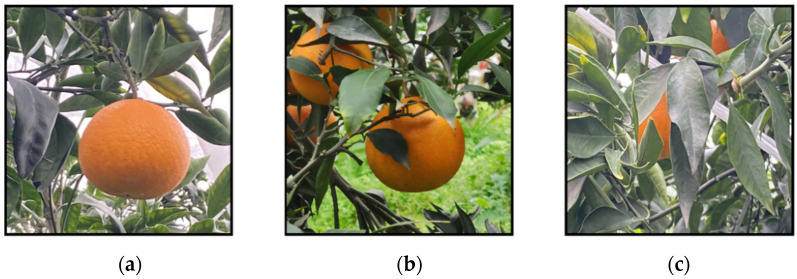

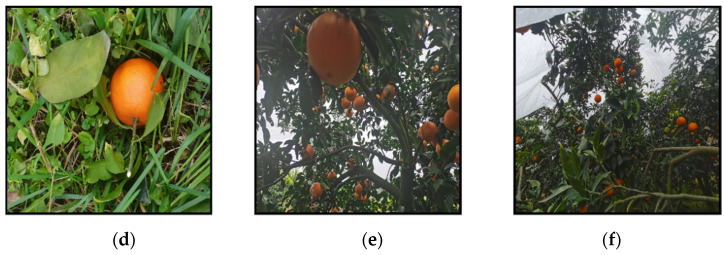
Part of the dataset is shown. (**a**) Single fruit; (**b**) Multiple fruits; (**c**) High degree of occlusion; (**d**) Different background conditions; (**e**) Low light; (**f**) Dense fruit aggregation.

In this experiment, we produced a dataset of citrus fruits in a citrus orchard setting. Images of citrus fruits were captured and collected using three Huawei NOVA7 mobile phones equipped with high-definition rear cameras. The maximum pixel value of the camera is 64 million, and the resolution of the fruit image is 4032 × 4032. The dataset was taken around November 2021, which is a good time for the citrus harvest. All images were acquired under natural daylight conditions when the outdoor environment was well-lit and warm. The location was in Danling County, Meishan city, Sichuan Province, which is a large county famous for citrus cultivation in China. The dataset includes four kinds of interference, namely overlap, occlusion, light-dark change, and distance change, which can best reproduce the conditions of citrus fruits as seen by human eyes in their natural environment. Part of the dataset is shown in Figure 4.

There are a total of 1266 citrus fruit images in the dataset, among which 6334 citrus fruits are captured and divided into a training set, a test set, and a validation set according to a ratio of 80/10/10. The training set consists of 1012 images, which contains 5338 citrus fruits, the test set consists of 127 images, which contains 507 citrus fruits, and the remaining 127 images contain 489 citrus fruits, which constitute the validation set. In addition, in the dataset about 40% of the single citrus images are smaller than 32 pixels × 32 pixels, which belong to the category of small target objects [26], about 20% of the citrus images are large target objects, and nearly 50% of the images include more than 3 citrus fruits. All the datasets were stored in JPG format. Table 1 shows the division of the datasets.

### 3.2. Data Preprocessing

To label the citrus fruit images in the dataset scientifically and reasonably, the following strategies were adopted in this experiment: the dataset was divided into four parts in advance, and four workers independently labeled the four parts. When the image could not be quickly identified as a citrus fruit, the image was extracted without labeling. Citrus fruits with occlusion degrees of more than 90% and less than 5 pixels were also not labeled. A fifth worker examined the labeled dataset. At the same time, the five staff members voted on how to label the unlabeled images by majority rule. The name of the labeling software used was LabelImg [30], the labeling box was rectangular, and the label name was set in Mandarin. The corresponding XML label file was generated, and the overall construction of the dataset was finally completed according to the COCO dataset [26].

**Figure 5:**
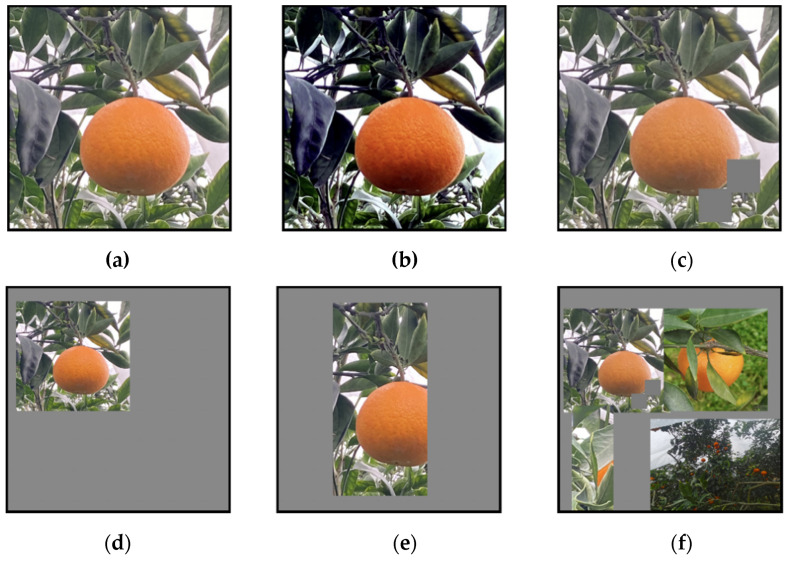
Example diagram of data enhancement. (**a**) Original image; (**b**) Contrast data enhancement; (**c**) Cutout data enhancement; (**d**) Scaling; (**e**) Random cropping; (**f**) Mosaic data enhancement.

In this experiment, a series of data enhancement operations were carried out on the produced dataset, including random cropping [31], adding noise [32], scaling [33], and random contrast adjustment [34]. The reasons for this data enhancement are as follows: there are various complex phenomena in the real environment, and the data enhancement operation can alleviate the network overfitting phenomenon to the greatest extent and enhance the robustness and generalization ability of the model. In addition, the YOLO network has its own Mosaic data enhancement method [22], which involves randomly cutting four images and stitching them into one image, which can increase the number of images for model training and thus improve the learnable content of the network. The specific data processing workflow is as follows: during the training process, HSV color space values are set as 0.015, 0.7, and 0.4 to enhance the hue, saturation, and brightness of the input image, and to alleviate the influence of occlusion, illumination, and shadow factors. Subsequently, the images were scaled with a random factor of 0.9, and each image was flipped horizontally with a probability of 0.5. Then, four processed images were taken out and a Mosaic jigsaw operation was carried out. The fixed area of the four images was captured in a matrix and finally spliced into a new picture to complete the combination of the picture and the object box. This method can enrich the background of the detected object and make the model not only focus on specific scenes to improve the generalization ability of the model, considering a citrus may appear on a branch, the ground, or on a desk. Some examples of image enhancement are shown in Figure 5.

### 3.3. Training Environment and Evaluation Indicators

The experiments in this paper are performed on a computer with the Ubuntu 18.04 operating system, which is configured with a 64-bit 2.20 GHZ twelve-core CPU, 32 GB of memory, an NVIDIA GeForce RTX A4000 GPU (NVIDIA Corporation, Stanta Clara, CA, USA), and 16 GB of video memory. The Compute Unified Device Architecture (CUDA) version is 11.0, the deep learning framework uses PyTorch 1.7.0, and the compiler is Python 3.8.

In the experimental model in this paper, the relevant hyperparameters are set as follows: the model receives images with a resolution of 640 × 640 pixels as a unified input, the initial learning rate of the model is 0.01, the momentum of the learning rate is 0.94, the optimizer adopts SGD [35], the weight decay value is 0.0005, and the training speed and video memory size are taken into account. The size of each training batch set in this paper is 8, and the number of training iterations of the model is uniformly set at 200 rounds.

To make the experiment objective, this paper evaluates the performance of the proposed method through a series of experiments. The definition of the evaluation index used is given as follows:

(6) $$\mathrm{Precision}=\frac{\mathrm{TP}}{\mathrm{TP}+\mathrm{FP}}$$

(7) $$\mathrm{Recall}=\frac{\mathrm{TP}}{\mathrm{TP}+\mathrm{FN}}$$

Among them, TP represents the number of citruses correctly identified, FP is the number incorrectly identified as citrus that is not citrus, and FN is the number not correctly detected as citrus. The precision rate refers to how much of the proportion of the objects detected by the model are correct citrus, and the recall rate refers to how much of the proportion of all the citruses are detected by the model.

Neither precision nor recall can comprehensively show the performance of the model, so the F1 score is used as a compromise between the two, as defined by Formula (8):

(8) $$F1=\frac{2}{\frac{1}{\mathrm{Precision}}+\frac{1}{\mathrm{Recall}}}=\frac{2\times \mathrm{Precision}\times \mathrm{Recall}}{\mathrm{Precision}\;+\;\mathrm{Recall}}$$

In Equation (9), AP refers to the area under the precision-recall curve (PR curve), and mAP is the average of APs of different classes. N is the number of classes of the test sample. Since there is only one category of citrus fruits in the dataset, N = 1.

(9) $$\mathrm{AP}=\int_{0}^{1}P\left(R\right)\mathrm{dR}$$

(10) $$\mathrm{mAP}=\frac{\sum_{1}^{N}\int_{0}^{1}P\left(R\right)\mathrm{dR}}{N}$$

### 3.4. The Proposed Citrus-YOLOv7 Model

The YOLOv7 version of the network model is used in this paper, and the relevant code can be found on GitHub [36]. The YOLOv7 network model mainly includes five components: input layer, backbone network, Neck, Head, and loss function.

In the input layer, three techniques of Mosaic data enhancement, adaptive anchor box calculation, and adaptive image scaling are adopted. As mentioned above, Mosaic data enhancement can sample four images and then randomly Mosaic, arrange, and crop them. In this paper, we believe that this input strategy can effectively solve the problem of small object detection. In the citrus dataset, the distribution of small objects and large objects in the sample is not uniform, and the distribution of small samples in the dataset can be increased by splicing and scaling, which makes the network more robust. Based on the initial anchor frame, the gap between the original anchor frame and the real frame is calculated through network training, and the optimal anchor frame parameters are obtained with a change of direction propagation. In this way, the rectangular frame with the appropriate ratio of length and width can be obtained according to the situation of the dataset, to improve the recall rate of the model. All the input images will be adaptively scaled to achieve the effect of normalization. The differences in the network model caused by the input images with different resolutions will be discussed in the following sections.

**Figure 6:**
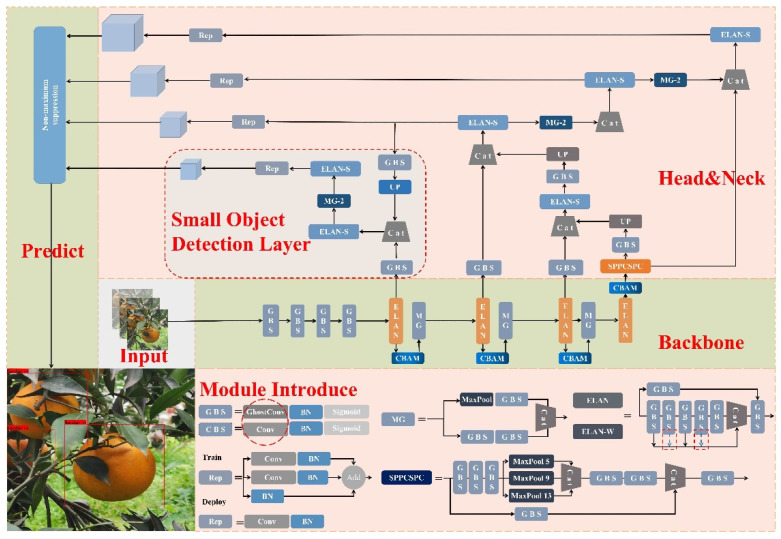
Citrus-YOLOv7 Network architecture.

The backbone network is a key part of feature extraction. The original YOLOv7 backbone network has a total of 50 modules, including the CBS module, ELAN module, and MP1 module. As shown in Figure 6, the improved backbone network has 54 modules, but the number of parameters is reduced by 50% compared with the original one. In this paper, the first improvement proposed is to replace the original CBS module with the GhostCBS module. The original CBS module consists of an ordinary convolution, a BN layer, and a SiLU. However, traditional convolution consumes a large amount of computing resources. To achieve lightweight deployment, the first step is to replace the original CBS module with the cheap Ghost convolution. Secondly, the original ELAN module is composed of six CBS modules; therefore, we also replace the CBS modules with the improved GhostCBS modules and add the CBAM attention module after the last GhostCBS module to form a new CG-ELAN module. In the backbone network, there are four CG-ELAN modules. The Traditional ELAN module is a highly efficient remote network. It can effectively extract the local structure of the image through shift convolution and reduce the model inference time through the shared attention mechanism. In the end, a CBAM module is introduced to make the original image. To enhance the effectiveness of the long-range self-attention of the network, while maintaining the number of parameters, the module takes into account both channel and spatial attention. The MP module consists of a Maxpool and a CBS, which are divided into MP1 and MP2. The difference between the two is that the channel of the former remains the same, while the channel of the latter is doubled. The same improvement is used to replace the CBS module with the Ghost-CBS module to enhance the sampling effect of the network.

The Head part of YOLOv7 combines the advantages of the feature pyramid network (FPN) and the path aggregation network (PAN) to form the PA-FPN structure, which makes the feature maps of different levels achieve the effect of efficient fusion. The first improvement made in this part of the paper is to replace all CBS modules with Ghost-CBS modules. Secondly, a small object detection layer is added, which can be used to generate a 160 × 160 feature map and broaden the detection range of the network. Although this method will bring a small increase in the amount of computation and detection speed, it can effectively improve detection accuracy. At the end of the Head, we use four RepConv modules that are not directly connected to reparameterize the network. The so-called re-parameterization here refers to the use of a multi-branch structure during training, which is equivalent to multiple neural networks participating in learning together, thereby improving the detection accuracy of the model. While re-parameterization is performed during inference to convert multiple networks into a single-head network, this is equivalent to multiple networks participating in a decision, which in turn leads to faster inference [37]. The reason why the non-directly connected structure is not adopted is that in other papers on YOLOv7, experiments have proven that the directly connected re-parameterized module will destroy the residual and splicing structure and generate gradient diversity for different feature maps [17], which is unfriendly towards the process of backpropagation.

In terms of loss function, YOLOv7 adopts a loss calculation method similar to YOLOv5, which is divided into object confidence loss, classification loss, and coordinate loss. Among them, the former two use a BCE cross-entropy loss. The coordinate loss makes use of the current excellent CIoU loss [38], which takes into account the overlapping area, center distance, aspect ratio, and other factors, and can further improve detection accuracy in solving the problem of non-overlapping detection boxes. The relevant formula is explained as follows.

Suppose S(x_n_) stands for Sigmoid function:

(11) $$S\left(x_{n}\right)=\frac{1}{1+e^{-x}}$$

The calculation formula of BCE cross-entropy loss is defined as follows, where w_n_ means to average the results and y_n_ represents the real sample label:

(12) $$L_{n}=-w_{n}\left[y_{n}\cdot \log S\left(x_{n}\right)+\left(1-y_{n}\right)\cdot \log\left(1-S\left(x_{n}\right)\right)\right]$$

The CIoU loss calculation formula is defined as follows, where IoU represents the intersection area of the prediction box and the real box:

(13) $$\mathrm{CIoU}=\mathrm{IoU}-\left(\frac{\rho^{2}\left(b,b^{\mathrm{gt}}\right)}{b^{2}}+\alpha \upsilon \right)$$

There are two notable parameters, υ and α, in Equation (13). The former is used to measure the consistency of the aspect ratio of the detection frame, and the latter is a trade-off parameter so that the overlap area factor can be given a higher regression priority.

(14) $$\upsilon =\frac{4}{\pi^{2}}{\left(\arctan\frac{w^{\mathrm{gt}}}{h^{\mathrm{gt}}}-\arctan\frac{w}{h}\right)}^{2}$$

(15) $$\alpha =\frac{\upsilon }{\left(1-\mathrm{IoU}\right)+\upsilon }$$

(16) $$L_{\mathrm{CIoU}}=1-\mathrm{CIoU}$$

**Figure 7:**
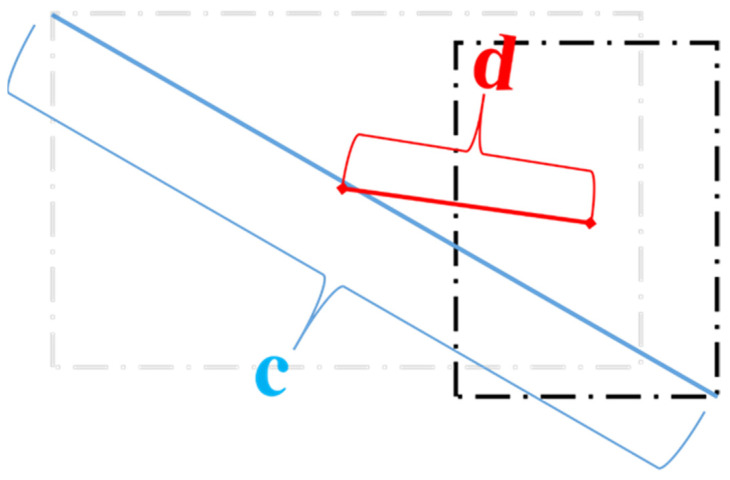
Illustration of CIoU loss formula.

The loss diagram for bounding box regression is shown in Figure 7 below, where d= $\rho^{2}\left(b,b^{\mathrm{gt}}\right)$ is the center point distance between the two bounding boxes, and c is the diagonal distance of the bounding box that can enclose at least the two boxes.

## 4. Results and Discussion

### 4.1. Comparison of the Overall Accuracy of Network Models

In this paper, relevant data are obtained through experiments. Firstly, the changes of mAP_@0.5_ and loss function are used as indicators to judge the training of the model, and the corresponding line graphs are drawn. mAP_@0.5_ refers to the AP value of the citrus detector when the threshold of IoU is set to 0.5. As the threshold of IoU increases gradually, the corresponding AP value will also decrease, so this is a reliable indicator. Loss of function as a means of observation model training model can reflect the existence of the overfitting phenomenon, when training loss value is very low, to verify that the loss value is large, and although many studies show that this is a fitting model, the model is just rote memorizing the data of the training set and does not achieve an ideal imitation of intelligence. If the training loss value and validation loss value are both large, it indicates that the model is underfitting, which reflects the lack of learning ability of the model.

**Figure 8:**
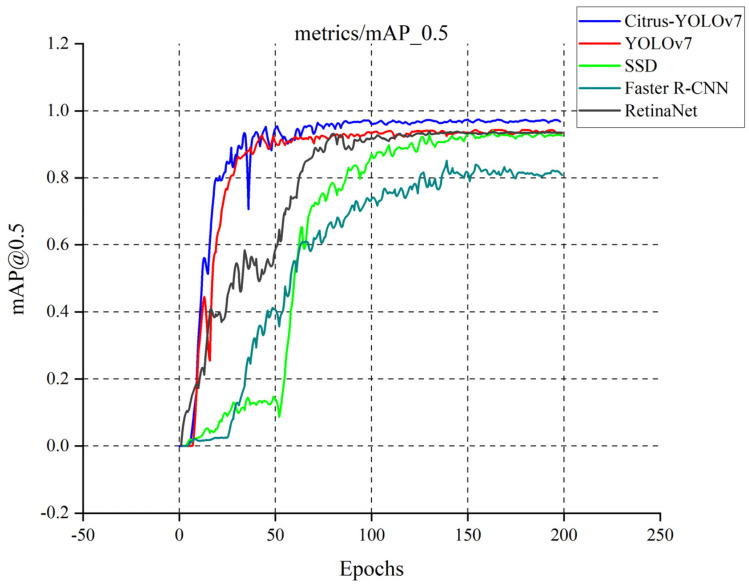
Accuracy variation of five object detectors.

**Figure 9:**
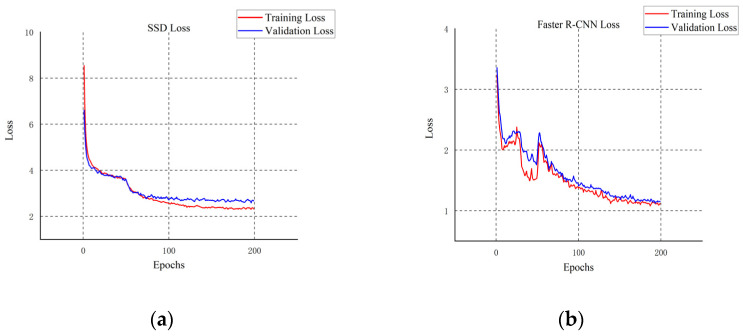
(**a**)The loss changes of the SSD model; (**b**)The loss changes of the Faster R-CNN model.

**Figure 10:**
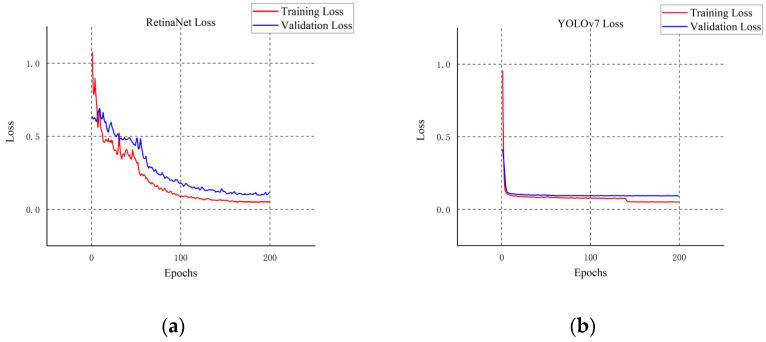
(**a**) The loss changes of the RetinaNet model; (**b**)The loss changes of the YOLOv7 model.

**Figure 11:**
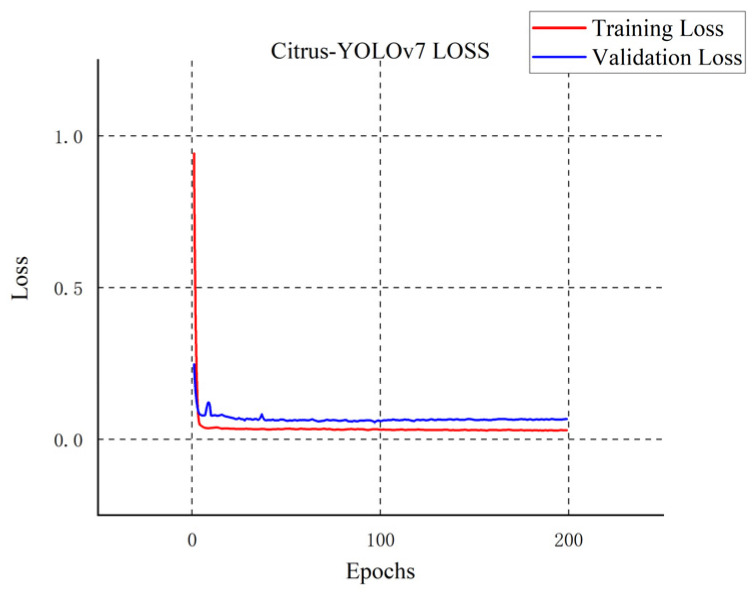
The loss changes of the Citrus-YOLOv7 model.

We compare the four most advanced object detectors with our improved model and draw a line chart with mAP_@0.5_ as the index. First of all, according to the five curves in different colors in Figure 8, the YOLOv7 series model has certain advantages in citrus detection, while the other SSD model [39], Faster R-CNN model [40], and RetinaNet model [41] have their defects in detection. As shown in Figure 9, Figure 10 and Figure 11, although the SSD model and RetinaNet model are close to the YOLOv7 series model in detection accuracy after 200 rounds of training, their convergence speed is slow and the early training fluctuates greatly. The Faster R-CNN model does not have an advantage in this problem, due to its network structure: because its RPN extraction network cannot produce a multi-layer fusion feature map, therefore it seems to be limited. The convergence speed of the improved Citrus-YOLOv7 model is significantly faster than that of other models in the training process. This is because the CBAM attention mechanism is added, which can enhance the feature distribution weight of the object to be detected in the spatial and channel dimensions and throw away the interference of useless features when fitting the results, and accelerate the convergence. At the same time, the mAP_@0.5_ value of the improved Citrus-YOLOv7 model is higher than that of the single-stage SSD model, the RetinaNet model, and the two-stage Faster R-CNN model, and is stronger than that of the YOLOv7 model before the improvement.

This paper compares and analyzes multiple indicators of object detection and the results are shown in Table 2. First, a quantitative analysis of mAP_@0.5_ was carried out. The Citrus-YOLOv7 model achieved a detection accuracy of 97.29%, which was 2.65% higher than the 94.64% of the YOLOv7 model achieved before the improvement, and was also higher than the third-ranked RetinaNet model. A result of 3.62% came out. On the harsh mAP_@[0.5:0.95]_ metric, Citrus-YOLOv7 is not inferior, achieving 74.83%. mAP_@[0.5:0.95]_ took the average AP value at different IoU thresholds. If the model result is only a simple frame of the object, which is far from the real frame, it will obtain poor results within this indicator. As can be seen from the table, because the resolution of the feature map generated by the Faster R-CNN model is low, and the RPN algorithm avoids generating overlapping boxes, it will filter out the boxes with low scores in the later stage of detection, which makes it prone to missed detection. Therefore, on the mAP_@[0.5:0.95]_ indicator, the score is 57.25%, which is a sharp drop. The score of YOLOv7 is 69.19%. This paper believes that part of the loss is caused by the missed detection of small target objects and large-area occluded objects. F1 can comprehensively evaluate the Precision and Recall indicators of the model. Citrus-YOLOv7 achieved 93.81% of the results here, which is nearly 1.27% higher than YOLOv7, 2.15% higher than RetinaNet, and 6.03% higher than SSD, and achieved a balanced performance in the Precision and Recall indicators effect. To sum up, the Citrus-YOLOv7 model proposed in this paper has a good recognition effect and superior detection performance on citrus fruits.

**Figure 12:**
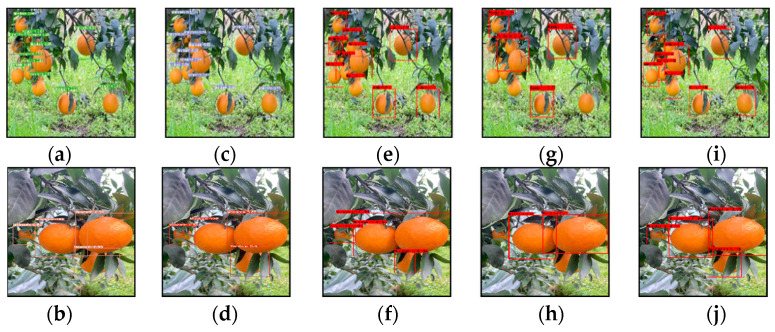
Test results of five models. (**a**,**b**) Citrus-YOLOv7; (**c**,**d**) YOLOv7; (**e**,**f**) Faster R-CNN; (**g**,**h**) SSD; (**i**,**j**) RetinaNet.

To understand the performance of the model more intuitively, the following Figure 12 shows the detection effect of the five models on two pictures randomly extracted from the test set. The superiority of the Citrus-YOLOv7 model can be seen directly from the specific results. Although the detection situation of YOLOv7 is roughly the same as that of the Citrus-YOLOv7 model, it has missed the detection of a small number of small target citrus, and the improved Citrus-YOLOv7 has enhanced its perception of small objects by adding a target frame for small object detection, which makes up for the shortcomings of the YOLOv7. At the same time, the average confidence of YOLOv7 is lower than that of the improved Citrus-YOLOv7 model, and the addition of the CBAM attention module after the improvement can improve confidence in the object. Although the RetinaNet model has high detection confidence, it is badly missed for citruses with a large degree of occlusion. SSD and Faster R-CNN have the same badly missed detection rates, even missing some obvious citrus fruits. Faster R-CNN also identified two citrus fruits as one object, which indicates that the two models are not suitable for the detection task of such datasets.

The paper also found a dataset on Kaggle called “Fruit Images for Object Detection” [42]. This data contains images of a variety of fruits, including citruses with different levels of occlusion and citruses in a real orchard environment. Therefore, it is considered a generalized test dataset. Firstly, the dataset with labels was downloaded and examined, and then augmented with data, resulting in a final dataset of 1000 images. In the end, the proposed model achieved 99.37% for mAP_@0.5_ and 83.8% for mAP_@[0.5:0.95]_ on this dataset, which indicates that the model can be widely applied to other similar datasets with a similar performance.

### 4.2. Comparison of Network Model Detection Speed

To confirm that the improved model in this paper can achieve faster detection speed to meet the requirements of hardware deployment detection, it is compared with the advanced object detector Faster R-CNN model, RetinaNet model, SSD model, and the YOLOv7 model on the test dataset. The specific experimental structure can be seen in Table 3. Because the model adopts GhostConv convolution to simplify the complex operations of traditional convolution, the average time of prediction of the Citrus-YOLOv7 reaches 69.28 ms, which is 1.31 ms shorter than that of the RetinaNet model and 125.79 ms shorter than that of the Faster R-CNN. It is even 8.89 ms shorter than the original YOLOv7, which will further demonstrate its advantages in large-scale video detection. In addition, in terms of it lightweight model, Citrus-YOLOv7 only requires 24.26 M parameters, it is only 0.64 M higher than that of the SSD model, and the calculation amount is only 76.40 g, which is 196.77 g smaller than that of the SSD model, 293.32 G lower than that of the highest Faster R-CNN, and 28.71 G less than the original YOLOv7, which can be accepted by most hardware devices. The improved Citrus-YOLOv7 model adopts an innovative network structure, which not only ensures the improvement of detection accuracy but also realizes the lightweight structure of the model.

### 4.3. Ablation Experiment

This part of the experiment explored the impact of the three improved methods on the network model. The plotted data are shown in Table 4. We carried out eight groups of experiments, adding different modules respectively, and compared them with the original YOLOv7 model using mAP_@0.5_, F1, Parameter, and Inference Time as indicators to measure. For convenience, the YOLOv7 model with a small object detection layer is named YOLOv7 + SD, the YOLOv7 model with GhostConv convolving is named YOLOv7 + GC, and finally, the network with the CBAM attention module is named YOLOv7 + CBAM, and so on.

As shown in the table, adding a small object detection layer and CBAM attention module to YOLOv7 can slightly improve the detection accuracy of the network, which is 0.41% and 0.52% higher than that of the YOLOv7 model, respectively, but the F1 index does not improve significantly. At the same time, we found that when the CBAM module was added to the network, the reasoning speed of YOLOv7 could be effectively improved to 53.87 ms, which was 11.68 ms lower than in the original version. If the original ordinary convolution is replaced by GhostConv convolution in YOLOv7, the number of parameters and reasoning time of the model can be greatly reduced, and the results of 24.89 M and 53.87 ms are achieved. In addition, through the pairwise combination of modules, as can be seen in the table, the combination of SD + CBAM modules has the highest accuracy improvement of the model, with a score of 93.53% in the F1 index, which is also a good performance. The combination of GC + CBAM modules can significantly reduce the reasoning time of the model, reaching 52.04 ms, and the minimum number of parameters of the model is 23.98 M. However, the introduction of the SD module will increase the number of parameters and reasoning time, but this paper believes that the combination of the three modules is a good match. Benefiting from the GhostConv module and CBAM module, it brings the performance of accelerated reasoning and lightweight deployment to the model, and the added small object detection layer and CBAM module bring a qualitative improvement to its detection accuracy. In summary, although our method sacrifices a small part of reasoning time, it brings significant improvements in accuracy, which is a worthwhile improvement.

## 5. Conclusions

Based on research on the state-of-the-art YOLOv7 model, this paper proposes a Citrus-YOLOv7 model, which is mainly used to solve the problem of detecting citrus in citrus orchards. There are three main improvement methods for the model. The first is to replace the traditional convolution module with the lightweight GhostConv convolution module. To achieve multi-scale feature fusion, the second is to add a small target detection layer, which can alleviate the fact that the original detection layer of YOLOv7 cannot adapt to small target objects. Finally, by imitating the human visual attention learning mechanism, the CBAM attention module that takes into account channel and spatial dimensions is used to complete the reorganization and optimization of the feature extraction and detection parts of the YOLOv7 backbone network, Neck, and Head. The Citrus-YOLOv7 model proposed in this paper has achieved excellent results in multiple indicators in a comparison of four target detectors on the test dataset. The mAP_@0.5_ value of this model is 97.29%, which is 14.36%, 3.62%, 4.48%, and 2.65% higher than the state-of-the-art Faster R-CNN, RetinaNet, SSD, and YOLOv7 models, respectively. On the mAP_@[0.5:0.95]_ indicator, the improved model achieved 74.83%, which is about 5.64% higher than the YOLOv7, and nearly 1.27% higher than the YOLOv7 in the F1 indicator, which is a good balance of Precision and Recall, therefore, it is a model with superior detection performance. In terms of detection speed and lightweight, the Citrus-YOLOv7 model achieves an average detection speed of 69.38 ms, which is lower than YOLOv7’s 78.27 ms. The parameter amount is nearly 0.64 M lower, and the calculation amount is saved by 28.71 G. It can be seen that the improved model in this paper achieves a degree of balance in accuracy, speed, and lightweight deployment, and can be used for the object detection of citrus fruits in a real orchard environment. In future work, we can continue to improve other module structures of the model, for example, context learning can be performed to make full use of target-related information in images and enhance the model’s ability to adapt to different scenarios.

## Figures and Tables

**Table 1:** The partitioning of the dataset.

	Name	Proportion	Number of Pictures	Number of Fruits
dataset	training set	80%	1012	5338
	validation set	10%	127	489
	test set	10%	127	507
total		100%	1266	6334

**Table 2:** Model performance table under multiple indicators.

Models	mAP_@0.5_	mAP_@[0.5:0.95]_	Precision	Recall	F1
Faster R-CNN	82.93%	57.25%	69.34%	90.24%	78.42%
RetinaNet	93.67%	67.90%	90.77%	92.56%	91.66%
SSD	92.81%	65.46%	86.12%	89.50%	87.78%
YOLOv7	94.64%	69.19%	93.69%	91.41%	92.54%
Citrus-YOLOv7	97.29%	74.83%	94.25%	93.37%	93.81%

**Table 3:** Comparison of prediction speed and computing resources.

Models	Inference Time	Parameter	FLOPs
Faster R-CNN	195.17 ms	136.69 M	369.72 G
RetinaNet	70.69 ms	36.33 M	145.34 G
SSD	91.02 ms	23.62 M	273.17 G
YOLOv7	78.27 ms	35.47 M	105.11 G
Citrus-YOLOv7	69.38 ms	24.26 M	76.40 G

**Table 4:** Ablation experiments of modules.

Methods	mAP_@0.5_	F1	Parameter	Inference Time
YOLOv7	94.64%	92.54%	35.47 M	73.27 ms
YOLOv7 + SD	95.05%	92.12%	36.00 M	74.31 ms
YOLOv7 + GC	92.73%	90.43%	24.89 M	53.87 ms
YOLOv7 + CBAM	95.16%	92.62%	34.31 M	61.59 ms
YOLOv7 + SD + GC	95.86%	92.08%	25.21 M	57.53 ms
YOLOv7 + GC + CBAM	96.63%	91.97%	23.98 M	52.04 ms
YOLOv7 + SD + CBAM	97.07%	93.53%	34.79 M	66.25 ms
YOLOv7 + SD + GC + CBAM	97.29%	93.81%	24.26 M	69.38 ms

## Data Availability

The data in this study are available on request from the corresponding author.
